# Data analysis of zoonoses notifications in Aboriginal and Torres Strait Islander populations in Australia 1996–2021: implications for One Health

**DOI:** 10.3389/fpubh.2023.1175835

**Published:** 2023-10-12

**Authors:** Tamara Riley, Raymond Lovett, Bonny Cumming, Anna Meredith, Neil E. Anderson, Joanne Thandrayen

**Affiliations:** ^1^National Centre for Epidemiology and Population Health, The Australian National University, Canberra, ACT, Australia; ^2^Animal Management in Rural and Remote Indigenous Communities (AMRRIC), Darwin, NT, Australia; ^3^The Royal (Dick) School of Veterinary Studies and The Roslin Institute, University of Edinburgh, Roslin, United Kingdom

**Keywords:** Aboriginal and Torres Strait Islander, animal health, environment, One Health, public health, zoonoses

## Abstract

**Introduction:**

Zoonoses are a health concern for Aboriginal and Torres Strait Islander peoples in Australia that face elevated risk of disease related to the environment and animals. Internationally, One Health is encouraged to effectively manage zoonoses by taking integrated approaches involving animal, human, and environmental health sectors to improve health outcomes. However, Australia’s health systems manage zoonotic diseases in animals and people separately which does not support a One Health approach. For the effective management of zoonoses, a strong evidence base and database regarding the epidemiology of zoonotic pathogens is needed. However, we currently lack this evidence limiting our understanding of the impact of zoonoses on Aboriginal and Torres Strait Islander populations.

**Methods:**

As a first step towards building the evidence base, we undertook a descriptive analysis of Aboriginal and Torres Strait Islander zoonotic notifications in Australia from 1996 to 2021. We presented notifications as annual notification rates per 100,000 population, and percentages of notifications by state, remoteness, sex, and age group.

**Results:**

Salmonellosis and campylobacteriosis were the most notified zoonoses with the highest annual notification rates of 99.75 and 87.46 per 100,000 population, respectively. The north of Australia (Queensland, Northern Territory and Western Australia), remote and outer regional areas, and young children (0–4 years of age) had the highest percentages of notifications.

**Discussion:**

To our knowledge, these findings are the first national presentation of the epidemiology of zoonoses within Aboriginal and Torres Strait Islander populations. A greater understanding of transmission, prevalence and impact of zoonoses on Aboriginal and Torres Strait Islander peoples (including animal and environmental health factors) is required to inform their effective management through a One Health approach.

## 1. Introduction

Zoonotic pathogens are a global health concern and can cause a threat to human health particularly where animals and humans live closely together. Zoonoses are diseases caused by pathogens that can be transmitted between animals and people through various avenues including airborne, vectors, direct or indirect contact, food borne, water borne, and soil borne transmission (1). Both domestic and wild animals can be involved in the transmission of zoonotic pathogens, with environmental exposure, such as via vectors, also a key component in many situations. Therefore, understanding relationships and interactions between animal, human, and environmental health is integral to understanding transmission pathways and addressing the risk of zoonoses.

Globally, there has been an increase in emerging zoonotic diseases with 60% of all infectious diseases in humans and 75% of emerging infectious diseases in humans of zoonotic origin and commonly originating from wildlife (2, 3). An example of an emerging infectious disease is SARS-CoV-2 which is responsible for the recent COVID-19 pandemic and is hypothesised to have originated from wild animals with the impacts on health felt globally (4, 5). Following an increase in zoonotic outbreaks, strong support for countries to adopt integrated health approaches has been recommended including focusing on environmental and animal management as part of public health responses to control disease (6, 7). Endemic diseases (including neglected zoonotic diseases) are also a great concern for low resourced communities, many of which have high Indigenous populations and can be at higher risk of zoonoses (8). Whilst zoonotic diseases are a risk for Indigenous communities, they are also among the most under-diagnosed diseases in humans with the full burden of disease not well understood (8).

Aboriginal and Torres Strait Islander peoples consist of an estimated 984,000 people across some 300 different language groups, making up approximately 3.8% of the Australian population (9). Whilst improvements in Aboriginal and Torres Strait Islander health are a focus of national policy within Australia (10), there continue to be large inequities in health outcomes and access to health care (11). Aboriginal and Torres Strait Islander peoples are disproportionately affected by diseases related to environmental health, including communicable diseases, with further disparities experienced in remote areas (12, 13). Environmental health factors contributing to this include quality of housing, water, air, sanitation, disease control, and food and water safety (12). The impact of these factors is hypothesised to increase due to the changing climate, which in tropical regions of Australia may lead to a range of negative sequalae including increased risk of vector borne zoonotic pathogens (14). Animal and human health factors, along with social determinants of health, also contribute to these disparities, for example remote areas have higher domestic animal populations with people and animals living closely together, many without access to animal health care or associated services (15–18). Communities in the north of Australia are at increased risk of exotic zoonotic pathogens that are present in neighbouring countries with surveillance vital to detection and disease control (19). Interactions with wild and feral animals can also increase risk of disease, for example hunting can be common practise in some communities increasing interactions between people, domestic, and wild animals (20). Due to the elevated risk of zoonoses in communities, animal health programmes can be beneficial in improving health outcomes and increasing awareness (21, 22), however, how to operationalise a One Health approach needs further consideration.

Within Australia, nationally notifiable diseases in people are those that have been assessed as a public health priority and meet multiple assessment criteria, including importance for Indigenous health (23). Nationally notifiable diseases are reportable to state and territory health authorities, with data supplied to the National Notifiable Diseases Surveillance System (NNDSS). The NNDSS includes the surveillance of more than 50 communicable diseases of national public health importance and is managed by the Australian Government Department of Health and Aged Care, with oversight provided by the Communicable Diseases Network Australia (24). This surveillance system helps with the monitoring, detection, and control of communicable diseases and informs the coordination of outbreak responses (25). Notifiable diseases disproportionately affect Aboriginal and Torres Strait Islander people accounting for 8.4% of all notifications from 1991 to 2011 (26). Notifications among Aboriginal and Torres Strait Islander populations are, however, thought to be an underestimation with Indigenous status commonly under reported (26). Whilst many diseases included in the NNDSS are of animal origin, the database only includes disease that is diagnosed in people.

Internationally, the effective management of zoonoses is seen as a priority by leading health organisations and a One Health approach is strongly encouraged at a global, national, and local level (27). One Health is an interdisciplinary approach to health recognising that the “health of humans, domestic and wild animals, plants, and the wider environment (including ecosystems) are closely linked and inter-dependent” (pg 2, 28). Zoonotic programmes that take a One Health approach and involve multiple sectors have been found to be more effective in reducing disease than those within a single sector (28). Zoonotic programmes targeting specific diseases may also require additional management approaches due to differing transmission pathways. The One Health approach can assist in understanding and addressing the factors that lead to the increased risk of communicable disease within Aboriginal and Torres Strait Islander populations. One Health aligns with Aboriginal and Torres Strait Islander cultures and knowledge’s that recognise these integral health relationships and support holistic approaches to health care (29). The underlying principles of One Health highlight the need for equity between sectors, inclusion and engagement of communities, transdisciplinary approaches, and acknowledging the role of the environment in health (30). It can also assist with timely and effective public health responses, accurate decision making, accountability, and shared responsibilities and resources (31, 32).

The development of joint human and animal health systems has been recommended for effective management of zoonoses, including data sharing and integrated surveillance systems (8). However, many countries do not have adequate mechanisms in place for managing zoonoses across human and animal health sectors limiting the ability to prevent and control disease (8, 31). Australia’s human and animal health systems are managed separately with limited communication between sectors (33). Therefore, the management of zoonoses is managed separately with notifiable pathogens and subsequent disease not consistent between databases or between states and territories with some zoonoses nationally notifiable in people but not animals [such as Q fever (also known as coxiellosis)]. National notifiable disease lists commonly focus on zoonoses related to livestock and wildlife due to economic and trade implications however, there is limited surveillance of zoonotic pathogens related to companion animals with dogs and cats common in communities. Some zoonoses of public health importance are also not nationally notifiable in people and animals, such as *Strongyloides* however, Strongyloides Australia has recommended its addition to the national notifiable list due to high rates in Aboriginal communities (34).

Therefore, the current approach does not adequately account for the impact of animal and environmental factors that contribute to human health outcomes, limiting our ability to improve the management of zoonoses (35, 36). It also leads to challenges in the identification, prevention, and control of zoonotic pathogens. Australia’s health system could benefit from an integrated national system, assisting in timely detection and effective management of zoonoses (33). Examples of this can be seen internationally in Denmark and Canada where they have taken an integrated and continuous approach to monitoring antimicrobial resistance in animals and people, however it can be argued that these systems fall short of enacting a true One Health approach (37, 38). Indigenous governance and leadership in health systems is also commonly limited and needs further consideration to strengthen and inform disease management (39).

Zoonotic pathogens are of increasing concern globally and they are commonly under-reported and neglected, with many gaps in our understanding of them within Aboriginal and Torres Strait Islander populations. To address this and inform a One Health approach to the management of zoonoses, we initially undertook a scoping review regarding zoonoses in Aboriginal and Torres Strait Islander populations and found many gaps within the evidence base (40). This study builds on these findings by investigating the epidemiology of notifiable zoonoses within Aboriginal and Torres Strait Islander populations in Australia.

## 2. Materials and methods

This study was conducted with approval from the Australian Institute of Aboriginal and Torres Strait Islander Studies (AIATSIS) Research Ethics Committee (EO243-20210406) and was undertaken by an Aboriginal-led and multidisciplinary research team.

### 2.1. Study design

Due to the current systems limitations, it was not possible to perform an integrated data analysis using animal and human health datasets due to differences in data access processes, data fields, and Indigenous identifiers. Therefore we limited our focus to the NNDSS and reported zoonoses in people.

We utilised the NNDSS database to undertake a descriptive analysis of zoonotic notifications in Aboriginal and Torres Strait Islander populations from 1996 to 2021. We aimed to understand the characteristics of notifications in the population by time, location, and demographic factors. First, the NNDSS disease list was assessed for zoonoses by two authors independently (TR, BC) with zoonoses of interest discussed and agreed. The zoonoses of interest included those that actively transmit between animals and people and did not include those that have a zoonotic origin but are now maintained through human-to-human transmission. A data request was then made to the Australian Government Department of Health and Aged Care for data access of the NNDSS database from 1996 to 2021 (24). The data analysis plan was developed with input from three authors (TR, RL, and JT) and analysis undertaken by two authors (TR as main analyst, JT as secondary analyst).

### 2.2. Statistical analysis

We performed a descriptive analysis of the NNDSS data from 1996 to 2021 to understand characteristics of zoonotic notifications within Aboriginal and Torres Strait Islander populations. The zoonoses included in the analysis were Ross River virus, brucellosis, campylobacteriosis, leptospirosis, listeriosis, ornithosis, Q fever, rabies, salmonellosis, viral haemorrhagic fever, Barmah Forest virus, Murray Valley encephalitis virus, Shiga toxin-producing *Escherichia coli* (STEC), anthrax, Japanese encephalitis virus, Kunjin virus, cryptosporidiosis, Australian bat lyssavirus, tularaemia, avian influenza, and Middle East respiratory syndrome. The variables used in the analysis included notification date received, state, statistical area level 3 (SA3s), disease code, age group, and sex for those notifications identified as Indigenous. Data were analysed in Stata 17 and Excel.

Aggregated data were used to calculate percentages of each zoonoses notified by Indigenous status nationally. We calculated notification rates per 100,000 Aboriginal and Torres Strait Islander population using aggregated annual notification data and estimated resident population data sourced from the Australian Bureau of Statistics (41). Notification rates were displayed graphically and zoonoses with rates less than 1 per 100,000 population removed. The notification rates were calculated as follows:


$$\text{Annual notification rate }=\left({\left[\text{number of cases notified}/\text{population size}\right]}^{*}\;100,000\right)$$

Aboriginal and Torres Strait Islander notifications are presented by state [Australian Capital Territory (ACT), New South Wales (NSW), Queensland (QLD), Western Australia (WA), Northern Territory (NT), Tasmania (TAS), Victoria (VIC), and South Australia (SA)], by sex (female, male) and by age group (0–4, 5–14, 15–29, 30–49, and 50+ years). Remoteness was derived using SA3s to group notification locations into the standard Australian Bureau of Statistics remoteness categories including major cities, inner regional, outer regional, remote and very remote (42). For state, remoteness, sex, and age group, percentages were calculated and displayed in bar charts. Missing data were included in results tables but were excluded from graphs.

## 3. Results

From 1996 to 2021, there were 29,786 Aboriginal and Torres Strait Islander zoonotic notifications, accounting for 3.1% of all zoonotic notifications in Australia. There were no notifications for rabies, viral haemorrhagic fever, anthrax, Australian bat lyssavirus, tularaemia, avian influenza, and Middle East respiratory syndrome.

Notifications were more common in Queensland (31.1%) and the Northern Territory (30.3%), and over half of all notifications were in remote (33.4%) and outer regional (29.9%) areas of Australia. The distribution of notifications in males and females was similar and those aged 0–4 years made up half of all notifications (50.8%; Table 1).

**Table 1:** Summary table of Aboriginal and Torres Strait Islander zoonoses notifications in Australia from 1996 to 2021.

Characteristics	Female *n* (%)	Male *n* (%)	Missing *n* (%)	Total *n* (%)
State				
ACT	48 (0.3)	48 (0.3)	0 (0.0)	96 (0.3)
NSW	1,739 (11.7)	1,956 (13.1)	4 (40.0)	3,699 (12.4)
NT	4,547 (30.7)	4,480 (30.0)	2 (20.0)	9,029 (30.3)
QLD	4,563 (30.8)	4,708 (31.5)	0 (0.0)	9,271 (31.1)
SA	717 (4.8)	705 (4.7)	0 (0.0)	1,422 (4.8)
TAS	68 (0.5)	58 (0.4)	0 (0.0)	126 (0.4)
VIC	267 (1.8)	269 (1.8)	2 (20.0)	538 (1.8)
WA	2,869 (19.4)	2,734 (18.3)	2 (20.0)	5,605 (18.8)
Total	14,818 (100.0)	14,958 (100.0)	10 (100.0)	29,786 (100.0)
Remoteness				
Major cities	2,170 (14.6)	2,246 (15.0)	4 (40.0)	4,420 (14.8)
Inner regional	1,038 (7.0)	1,046 (7.0)	2 (20.0)	2,086 (7.0)
Outer regional	4,382 (29.6)	4,521 (30.2)	3 (30.0)	8,906 (29.9)
Remote	4,974 (33.6)	4,971 (33.2)	1 (10.0)	9,946 (33.4)
Very remote	1,376 (9.3)	1,417 (9.5)	0 (0.0)	2,793 (9.4)
Missing	878 (5.9)	757 (5.1)	0 (0.0)	1,635 (5.5)
Total	14,818 (100.0)	14,958 (100.0)	10 (100.0)	29,786 (100.0)
Age group				
0–4 years	6,980 (47.1)	8,147 (54.5)	2 (20.0)	15,129 (50.8)
5–14 years	1,044 (7.1)	1,216 (8.1)	0 (0.0)	2,260 (7.6)
15–29 years	2,091 (14.1)	1,872 (12.5)	4 (40.0)	3,967 (13.3)
30–49 years	2,647 (17.9)	2,100 (14.0)	2 (20.0)	4,749 (15.9)
50+ years	2,053 (13.9)	1,618 (10.8)	2 (20.0)	3,673 (12.3)
Missing	3 (0.02)	5 (0.03)	0 (0.0)	8 (0.03)
Total	14,818 (100.0)	14,958 (100.0)	10 (100.0)	29,786 (100.0)

**Figure 1:**
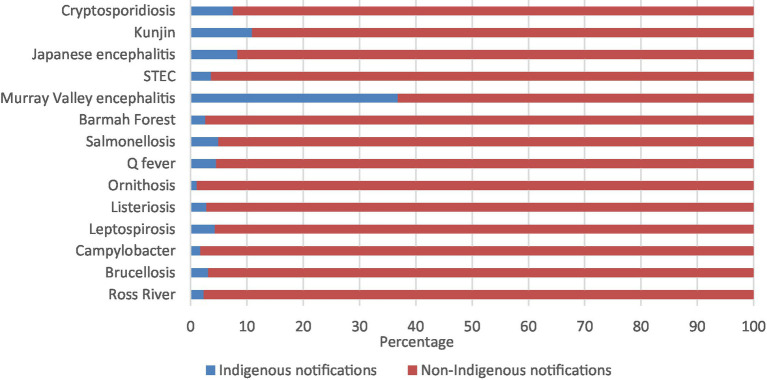
Percentage of zoonoses notifications by Indigenous status 1996–2021.

The zoonoses with the highest percentage of Aboriginal and Torres Strait Islander notifications were Murray Valley encephalitis (36.8%), followed by Kunjin virus (10.9%), Japanese encephalitis virus (8.3%), and cryptosporidiosis (7.5%; Figure 1; Supplementary Table 1).

**Figure 2:**
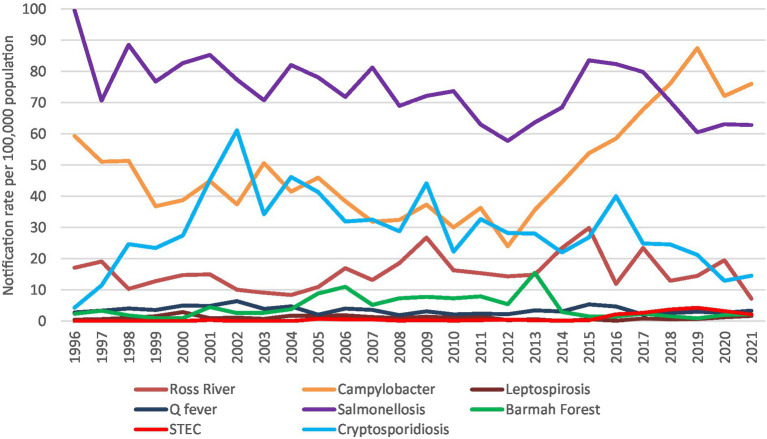
Aboriginal and Torres Strait Islander zoonoses notification rates per 100,000 population 1996–2021.

The zoonoses with the highest annual notification rates (per 100,000 population) were salmonellosis (99.75), campylobacteriosis (87.46), cryptosporidiosis (61.14), Ross River virus (29.85), Barmah Forest virus (15.45), Q fever (6.39), STEC (4.25), and leptospirosis (2.88). All other diseases had annual rates of 1 or less per 100,000 population (Figure 2; Supplementary Table 2).

**Figure 3:**
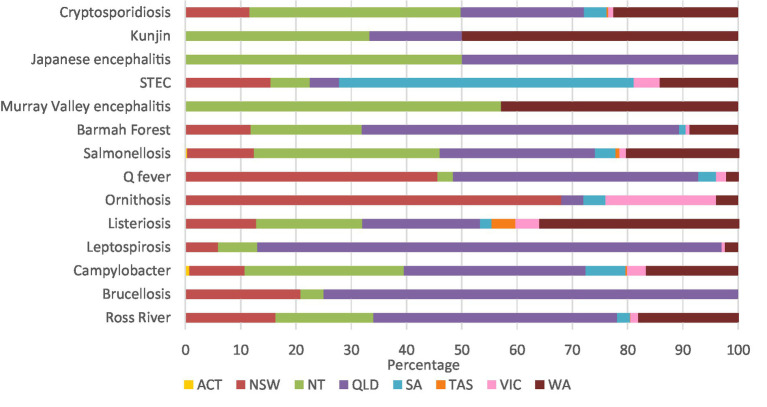
Aboriginal and Torres Strait Islander zoonoses notifications (%) by state 1996–2021.

Leptospirosis (84.0%), brucellosis (75.0%), and Barmah Forest virus (57.4%) had high percentages of notifications in Queensland. Similarly, 68.0% of ornithosis cases were in New South Wales, 57.1% of Murray Valley encephalitis cases were in the Northern Territory, and 53.3% of STEC cases were in South Australia. Half of all Kunjin virus cases were in Western Australia, and half the Japanese encephalitis cases were in the Northern Territory and Queensland (Figure 3; Supplementary Table 3A).

**Figure 4:**
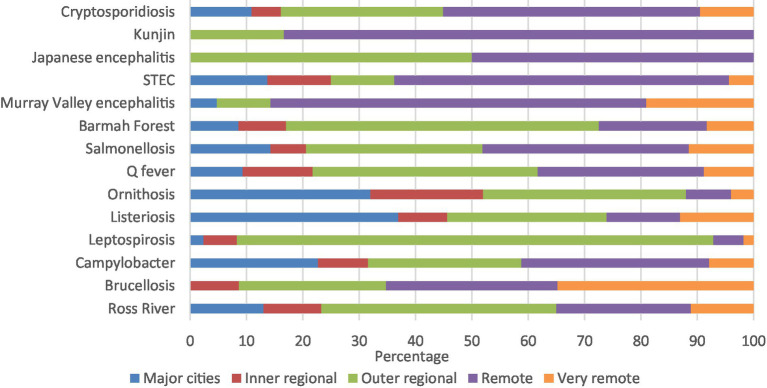
Aboriginal and Torres Strait Islander zoonoses notifications (%) by remoteness 1996–2021.

Kunjin virus (83.3%), Murray Valley encephalitis (66.7%), and STEC (56.2%) had high percentages of notifications in remote areas. Similarly, majority of leptospirosis (84.0%) and Barmah Forest virus (54.2%) were in outer regional areas, with half the Japanese encephalitis notifications in remote areas and half in outer regional areas (Figure 4; Supplementary Table 3B).

**Figure 5:**
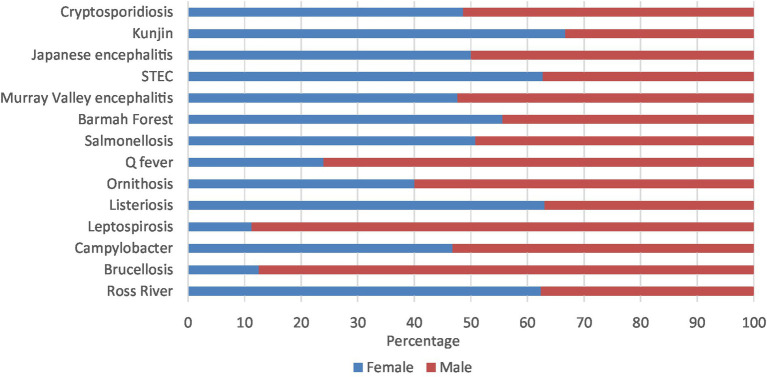
Aboriginal and Torres Strait Islander zoonoses notifications (%) by sex 1996–2021.

Leptospirosis (88.8%), brucellosis (87.5%), Q fever (76.1%), and ornithosis (60.0%) had majority of notifications in males. On the other hand, Kunjin virus (66.7%), STEC (62.7%), Ross River virus (62.4%), and listeriosis (61.7%) had majority of notifications in females. The other zoonoses presented in similar percentages in both males and females (Figure 5; Supplementary Table 3C).

**Figure 6:**
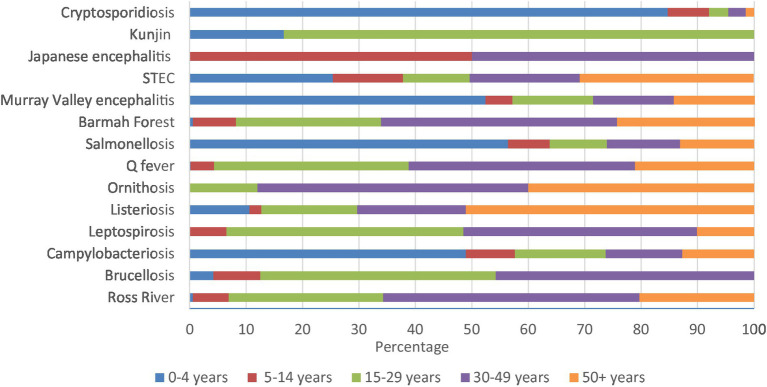
Aboriginal and Torres Strait Islander zoonoses notifications (%) by age group 1996–2021.

Cryptosporidiosis (84.7%), salmonellosis (56.4%), Murray Valley encephalitis (52.4%), and campylobacteriosis (48.9%) had high percentages in 0–4 years. Similarly, majority of Kunjin virus (83.3%) were in 15–29 years, half of Japanese encephalitis were in 5–14 and 30–49 years, and 51.1% of listeriosis notifications were in 50+ years of age (Figure 6; Supplementary Table 3D).

## 4. Discussion

### 4.1. Summary results

Just over 3% (3.1%) of all zoonotic notifications reported to the NNDSS from 1996 to 2021 were reported to be Aboriginal and Torres Strait Islander people. The percentage of Aboriginal and Torres Strait Islander zoonotic notifications was broadly similar to the overall population of 3.8% (9). However, Indigenous status is commonly underreported with a historical analysis identifying over half of all notifications to the NNDSS do not report Indigenous status (43). As these notifications are included as non-Indigenous, Aboriginal and Torres Strait Islander zoonotic notifications are likely to be underrepresented. Also, the barriers in accessing health care in many communities limit the ability to understand the true impact of zoonoses.

The highest percentages of zoonotic notifications were Murray Valley encephalitis, followed by Kunjin virus, and Japanese encephalitis however, when looking at annual notification rates all these diseases had rates less than 1 notification per 100,000 population per year. Alternatively, the zoonoses with the highest annual notification rates were salmonellosis, campylobacteriosis, and cryptosporidiosis. The zoonoses that did not have any notifications were rabies, viral haemorrhagic fever, anthrax, Australia bat lyssavirus, tularaemia, avian influenza, and Middle East respiratory syndrome.

Notifications were broadly in line with the percentage of the population in each state and territory with Queensland, Northern Territory, Western Australia, and New South Wales having the highest percentage of notifications and the Australian Capital Territory having the lowest (9). The most common remoteness category reported was remote areas of Australia which is a concern, as 18% of Aboriginal and Torres Strait Islander peoples live in remote and very remote areas (44) and these areas face higher risk of communicable diseases and limited access to health care (12, 13).

The percentage of total notifications by sex was similar. There are occupational exposures related to some zoonoses such as leptospirosis, brucellosis, and Q fever, all of which were more common for males and common exposures can include working with animals and animal products (45). Over half of all notifications were for children aged 0–4 years, with cryptosporidiosis and salmonellosis common in this age group. Whilst these zoonoses were the most common, they can also present with vague clinical signs and may not be identified therefore, results may be underrepresented. The Aboriginal and Torres Strait Islander population has a young age structure with one-third of the population under 15 years of age therefore, a higher level of notifications in young people would be expected (9).

The commonality of zoonoses notified for the north of Australia, for remote areas, and for young children may be due to an increased exposure to vectors and animals that carry and transmit disease. The north of Australia faces a tropical climate with vectors, such as mosquitos, common and the risk of vector borne zoonoses likely to increase due to a changing climate (14). Remote areas also have higher domestic animal populations and increased exposure where people and animals live closely together, many without access to animal health care or associated services (18). Therefore, the risk of zoonoses to people living in these areas may be higher. Similarly, communities can face increased environmental exposures due to a lack of appropriate housing and infrastructure, sanitation facilities, air quality, and food and water sources, increasing the risk of transmission and disease (12, 46). The most common zoonoses notified are usually food borne, highlighting the importance of appropriate food handling, storage, and food security, with food insecurity disproportionately experienced in Aboriginal and Torres Strait Islander communities, particularly in remote areas (47). Another factor that may explain higher notifications in remote areas is hunting activities which exposes people and domestic animals to wild animals that can carry disease (20).

These findings highlight that improving zoonoses prevention strategies within Aboriginal and Torres Strait Islander populations using One Health approaches, particularly for the north of Australia, for remote areas, and for young children, should be prioritised to reduce zoonotic notifications in the population. It is also important to consider zoonoses in the overall context of burden of disease within Aboriginal and Torres Strait Islander populations. To enact a One Health approach, strategies should acknowledge and address all One Health sectors and increase awareness about the transmission and risk of zoonoses including animal and environmental health exposures.

### 4.2. Strengths and limitations

To our knowledge, this is the first comprehensive national study of Aboriginal and Torres Strait Islander zoonotic notifications. This project was undertaken by an Aboriginal-led multidisciplinary research team with findings contributing to our understanding of zoonoses in the population whilst also highlighting gaps in the current system. The findings have highlighted areas of high notifications including specific diseases (salmonellosis and campylobacteriosis), young children (0–4 years of age), remote and outer regional areas, and the north of Australia (Queensland, Northern Territory, Western Australia). Additionally, we have identified gaps in our understanding of the true impact of zoonoses on the population and subsequent management.

Due to the current systems limitations, we reduced our focus to the NNDSS and reported zoonoses in people. This limited our ability to analyse the animal and environmental health factors that contribute to zoonotic notifications in people. Also, as we focused on notifiable zoonoses, we were not able to analyse zoonoses that may be prevalent in the population but are not considered notifiable. In interpreting the data, it is critical to recognise that notification data are based on diagnostic testing therefore, areas facing limited health care and laboratory diagnostic capacity may be underrepresented (10). Animals can also be sub-clinical carriers of zoonotic pathogens and not show signs of disease therefore, without monitoring, surveillance, and improved diagnostic capacity in animal’s zoonotic pathogens may not be identified. Changes in national surveillance case definitions, laboratory testing methods, and policies regarding collection of Indigenous status information may have impacted on the results however, this was outside the scope of this study.

The Indigenous notifications analysed relied on an Indigenous identifier being collected which is commonly underreported (26). Despite original intentions, we were also not able to analyse severity of disease (including health outcomes such as hospitalisations and deaths), or exposure factors due to large amounts of missing data. This limited our understanding of the animal and environmental exposures and subsequent transmission pathways, with evidence of zoonotic transmission between animals and people unconfirmed. Evidence has found that data completeness, timeliness, and inflexibility of the NNDSS database is problematic, with multiple stakeholders at state, territory, and national levels involved in its management (48). Therefore, improved data collection processes that consider the collection of Indigenous identifiers and involve multiple sectors should be considered to improve data completeness, accuracy of analyses, and inform public health responses (43).

### 4.3. Implications

Implementing integrated systems that involve multiple health sectors could assist with effective management of zoonoses and is an aim of the One Health approach, yet, systems in Australia do not currently facilitate this (33). International examples of joint systems can be seen in relation to antimicrobial resistance, however these systems need further development to truly enact a One Health approach. There is also need to incorporate meaningful inclusion of Indigenous Peoples through developing strong networks and governance structures that promote Indigenous leadership and engagement (39). These collaborative approaches can help to address public health risks at the animal-human-environment interface, improving the prevention and control of zoonoses (49). A One Health Framework can be adopted to prevent and control disease with the collaboration of the animal, human and environmental health sectors likely to be more effective than programmes in a single sector (28). Consistency in the management of zoonoses between health sectors has also been recommended internationally including standardised case definitions and notifiable disease lists for both animals and people, and a coordination centre for reporting and sharing data on zoonotic pathogens and subsequent disease (50). Strengthening communication between sectors including consistency of terminology and training related to zoonoses, and the development of a national One Health plan for addressing zoonoses with shared priorities and responsibilities is also recommended (32, 50).

An existing criterion for communicable diseases to be determined a public health priority and classified as nationally notifiable is the pathogen’s importance to Indigenous health (23), therefore a strong evidence base and database is needed to understand the contribution of zoonotic pathogens to human disease (40). This is also needed to undertake a national zoonotic disease prioritisation process which could help to improve the management of zoonoses in Aboriginal and Torres Strait Islander populations. Examples of prioritisation criteria include severity of disease in humans, availability of prevention and control strategies, potential to cause an epidemic or pandemic in animals or people, and social and economic impacts (50). Whilst some of these criteria may not be relevant for developed countries that have lower prevalence of zoonoses nationally, they are important considerations for Aboriginal and Torres Strait Islander communities that face higher risk of communicable diseases (51).

The relationships and interactions between people, animals, and the environment needs further investigation within Aboriginal and Torres Strait Islander populations, particularly in areas with higher levels of zoonoses notifications. Whilst environmental exposures are integral to the transmission of zoonotic pathogens, environmental health is commonly underrepresented in Indigenous One Health research (29). Environmental health data will continue to be a priority as we see the effects of a changing climate on health outcomes. Australia is considering the development of a national Centre for Disease Control to address emerging and existing health risks and this may address some of the current gaps within the management of zoonoses (52). However, it is yet to be seen if a One Health approach will be supported and how the management of zoonoses within communities will be addressed. Integrated approaches to the management of zoonotic disease and support for Indigenous leadership and governance within the national system is called for to improve the management of zoonoses within Aboriginal and Torres Strait Islander populations.

Futures studies should consider multidisciplinary approaches and further analysis of specific diseases, including trends over time, to improve understanding of zoonoses. Future studies may also include examining the risk of disease related to the social determinants of health (including cultural considerations) (31) and the severity of disease however, this would require holistic data and improved data completeness. Importantly, research within this space should foster Aboriginal and Torres Strait Islander leadership and genuine community engagement, using a transdisciplinary approach, to strengthen partnerships and focus research priorities (53, 54).

### 4.4. Next steps

This study builds on findings from a zoonoses scoping review that found gaps in the evidence base regarding zoonoses and Aboriginal and Torres Strait Islander populations (40). It also found that despite the strong conceptual foundations of One Health, evidence is lacking in its application and there is a need for research, programmes, and policies that prioritise Aboriginal and Torres Strait Islander leadership, incorporate multiple health sectors, and focus on zoonoses through a One Health approach. These findings will be built on through the development of recommendations for the management of zoonoses in Aboriginal and Torres Strait Islander populations through a One Health approach.

A national integrated One Health system is supported globally and could benefit the management of zoonoses for Aboriginal and Torres Strait Islander populations in Australia. However, consideration of consistency and collaboration between health sectors in the prevention and control of zoonoses for effective management of disease is key. There is also a need for Aboriginal and Torres Strait Islander leadership and engagement in research, policy, and programmes to ensure Australia’s zoonotic disease management is effective and appropriate for the population. A continuing challenge is the need for effective partnerships and communication between animal, human and environmental health sectors in research and public health to adopt holistic community health approaches and improve the management of zoonoses.

## Data availability statement

The original contributions presented in the study are included in the article/Supplementary material, further inquiries can be directed to the corresponding author.

## Author contributions

TR, RL, BC, and JT: conceptualization and methodology. TR and JT: formal analysis and investigation. TR: writing—original draft preparation and project administration. TR, RL, BC, AM, NA, and JT: writing—review and editing. RL, BC, AM, NA, and JT: supervision. All authors contributed to the article and approved the submitted version.
