# Supplementary material for: Data analysis of zoonoses notifications in Aboriginal and Torres Strait Islander populations in Australia 1996–2021: implications for One Health
Source: Front Public Health. 2023 Oct 12;11:1175835. doi: 10.3389/fpubh.2023.1175835 (PMC10602743; doi:10.3389/fpubh.2023.1175835)
Supplement: Supplementary file 1 [file Table_1.pdf]

Supplementary Material

Table 1: National zoonoses by Indigenous status 1996-2021

Zoonoses	Indigenous cases (n)	Indigenous cases (%)	Non Indigenous cases (n)	Non-Indigenous cases (%)	Total (n)
Japanese encephalitis virus	2	8.3	22	91.7	24
Kunjin virus	6	10.9	49	89.1	55
Murray Valley encephalitis virus	21	36.8	36	63.2	57
Brucellosis	24	3.1	745	96.9	769
Ornithosis	25	1.1	2183	98.9	2208
Listeriosis	47	2.8	1649	97.2	1696
STEC	169	3.6	4585	96.4	4754
Leptospirosis	169	4.3	3730	95.7	3899
Q fever	568	4.5	12086	95.5	12654
Barmah Forest virus	727	2.6	27218	97.4	27945
Ross River virus	2637	2.3	112518	97.7	115155
Cryptosporidiosis	4779	7.5	59279	92.5	64058
Campylobacteriosis	8318	1.7	469524	98.3	477842
Salmonellosis	12251	4.9	237499	95.1	249750
Total	29743*	3.1	931123	96.9	960866

*Table is based on aggregated data which has slightly different total Indigenous cases

Table 2: Zoonoses notification rates per 100,000 population in Aboriginal Torres Strait Islander population nationally by year 1996-2021

Year	Ross River	Brucellosis	Campylobacteriosis	Leptospirosis	Listeriosis	Ornithosis	Q fever	Salmonellosis	Barmah Forest	Murray Valley encephalitis	STEC	Japanese encephalitis	Kunjin	Cryptosporidiosis	Total (n)
1996	17.09	0.00	59.38	0.43	0.21	0.21	2.78	99.75	2.35	0.00	0.00	0.00	0.00	4.27	873
1997	19.10	0.21	51.08	0.62	0.42	0.21	3.32	70.61	3.32	0.21	0.00	0.00	0.00	11.42	773
1998	10.31	0.20	51.33	1.01	0.40	0.00	4.04	88.52	1.82	0.20	0.00	0.20	0.20	24.65	905
1999	12.79	0.39	36.81	1.57	0.39	0.00	3.54	76.76	0.98	0.79	0.00	0.00	0.00	23.42	800
2000	14.77	0.00	38.74	2.88	1.34	0.19	4.99	82.67	0.96	0.96	0.00	0.00	0.38	27.43	914
2001	14.96	0.00	44.88	0.94	0.56	0.56	4.86	85.28	4.49	0.00	0.37	0.00	0.00	45.26	1081
2002	10.04	0.18	37.41	1.10	0.55	0.37	6.39	77.38	2.56	0.00	0.00	0.00	0.00	61.14	1080
2003	9.09	0.00	50.63	0.71	0.18	0.36	3.92	70.77	2.67	0.00	0.00	0.00	0.00	34.23	968
2004	8.36	0.35	41.46	1.74	0.17	0.35	4.70	82.06	3.83	0.17	0.00	0.00	0.17	46.17	1088
2005	10.89	0.51	45.96	1.70	0.17	0.34	2.04	78.13	8.85	0.17	0.68	0.00	0.00	41.36	1121
2006	16.96	0.33	38.41	1.83	0.00	0.50	3.99	71.83	10.97	0.00	0.50	0.00	0.17	31.92	1067
2007	13.16	0.16	31.85	1.30	0.16	0.00	3.58	81.26	5.20	0.00	0.49	0.00	0.00	32.50	1044
2008	18.60	0.32	32.42	0.95	0.16	0.00	1.91	68.98	7.31	0.00	0.16	0.00	0.00	28.77	1004
2009	26.75	0.16	37.32	1.40	0.31	0.00	3.11	72.16	7.78	0.16	0.31	0.00	0.00	44.16	1245
2010	16.29	0.00	30.00	1.07	0.30	0.15	2.13	73.70	7.31	0.00	0.15	0.00	0.00	22.23	1007
2011	15.38	0.00	36.28	1.49	0.30	0.00	2.39	63.00	7.91	0.60	0.30	0.00	0.00	32.69	1074
2012	14.33	0.15	23.97	0.29	0.29	0.15	2.19	57.74	5.41	0.00	0.44	0.00	0.00	28.21	911
2013	14.88	0.00	35.63	0.57	0.29	0.14	3.43	63.68	15.45	0.00	0.29	0.00	0.00	28.05	1135
2014	23.38	0.00	44.52	0.00	0.14	0.00	3.08	68.47	2.94	0.00	0.14	0.00	0.00	21.98	1176
2015	29.85	0.14	53.82	0.55	0.27	0.00	5.34	83.54	1.51	0.27	0.27	0.00	0.00	26.84	1478
2016	11.92	0.13	58.52	0.13	0.27	0.27	4.69	82.35	1.47	0.00	2.14	0.00	0.00	40.04	1508
2017	23.43	0.00	67.80	0.79	0.13	0.13	2.09	79.84	2.36	0.00	2.62	0.00	0.13	24.87	1560
2018	12.92	0.13	76.10	0.51	0.26	0.00	2.56	70.34	1.53	0.13	3.71	0.00	0.00	24.56	1507
2019	14.49	0.12	87.46	0.62	0.50	0.00	3.00	60.47	0.87	0.00	4.25	0.00	0.00	21.24	1545
2020	19.53	0.24	72.13	1.22	0.00	0.12	2.68	63.10	1.95	0.00	3.17	0.00	0.00	12.94	1451
2021	7.15	0.12	76.04	1.67	0.00	0.12	3.34	62.81	2.15	0.00	2.15	0.12	0.00	14.54	1428

Table 3: Zoonoses percentages by demographic characteristics in Aboriginal Torres Strait Islander population from 1996-2021

	Ross River	Brucellosis	Campylobacteriosis	Leptospirosis	Listeriosis	Ornithosis	Q fever	Salmonellosis	Barmah Forest	Murray Valley encephalitis	STEC	Japanese encephalitis	Kunjin	Cryptosporidiosis	Total
**a) State (%)**															
ACT	0.0	0.0	0.7	0.0	0.0	0.0	0.0	0.3	0.0	0.0	0.0	0.0	0.0	0.1	0.3
NSW	16.3	20.8	10.0	5.9	12.8	68.0	45.6	12.1	11.8	0.0	15.4	0.0	0.0	11.5	12.4
NT	17.7	4.2	28.8	7.1	19.2	0.0	2.8	33.6	20.1	57.1	7.1	50.0	33.3	38.2	30.3
QLD	44.1	75.0	32.9	84.0	21.3	4.0	44.4	28.1	57.4	0.0	5.3	50.0	16.7	22.3	31.1
SA	2.3	0.0	7.2	0.0	2.1	4.0	3.2	3.7	1.2	0.0	53.3	0.0	0.0	4.1	4.8
TAS	0.1	0.0	0.3	0.0	4.3	0.0	0.0	0.7	0.0	0.0	0.0	0.0	0.0	0.3	0.4
VIC	1.4	0.0	3.4	0.6	4.3	20.0	1.8	1.2	0.7	0.0	4.7	0.0	0.0	0.9	1.8
WA	18.2	0.0	16.7	2.4	36.2	4.0	2.3	20.5	8.8	42.9	14.2	0.0	50.0	22.6	18.8
Total	100.0	100.0	100.0	100.0	100.0	100.0	100.0	100.0	100.0	100.0	100.0	100.0	100.0	100.0	100.0
**b) Remoteness (%)**															
Major cities	12.4	0.0	21.4	2.4	36.2	32.0	9.2	13.5	8.4	4.8	13.0	0.0	0.0	10.3	14.8
Inner regional	9.8	8.3	8.4	5.9	8.5	20.0	12.2	6.0	8.3	0.0	10.7	0.0	0.0	4.9	7.0
Outer regional	39.8	25.0	25.6	84.0	27.7	36.0	39.1	29.5	54.2	9.5	10.7	50.0	16.7	27.1	29.9
Remote	22.7	29.2	31.4	5.3	12.8	8.0	28.9	34.5	18.7	66.7	56.2	50.0	83.3	43.0	33.4
Very remote	10.6	33.3	7.4	1.8	12.8	4.0	8.6	10.8	8.1	19.1	4.1	0.0	0.0	9.0	9.4
Missing	4.7	4.2	5.9	0.6	2.1	0.0	2.1	5.8	2.3	0.0	5.3	0.0	0.0	5.7	5.5
Total	100.0	100.0	100.0	100.0	100.0	100.0	100.0	100.0	100.0	100.0	100.0	100.0	100.0	100.0	100.0
**c) Sex (%)**															
Female	62.4	12.5	46.7	11.2	61.7	40.0	23.9	50.8	55.6	47.6	62.7	50.0	66.7	48.6	49.8
Male	37.6	87.5	53.2	88.8	36.2	60.0	76.1	49.2	44.4	52.4	37.3	50.0	33.3	51.4	50.2
Missing	0.0	0.0	0.05	0.0	2.1	0.0	0.0	0.02	0.0	0.0	0.0	0.0	0.0	0.04	0.03
Total	100.0	100.0	100.0	100.0	100.0	100.0	100.0	100.0	100.0	100.0	100.0	100.0	100.0	100.0	100.0
**d) Age group (%)**															
0-4	0.6	4.2	48.9	0.0	10.6	0.0	0.2	56.4	0.6	52.4	25.4	0.0	16.7	84.7	50.8
5-14	6.3	8.3	8.7	6.5	2.1	0.0	4.1	7.4	7.6	4.8	12.4	50.0	0.0	7.3	7.6

	Ross River	Brucellosis	Campylobacteriosis	Leptospirosis	Listeriosis	Ornithosis	Q fever	Salmonellosis	Barmah Forest	Murray Valley encephalitis	STEC	Japanese encephalitis	Kunjin	Cryptosporidiosis	Total
15-29	27.4	41.7	16.1	42.0	17.0	12.0	34.5	10.1	25.7	14.3	11.8	0.0	83.3	3.4	13.3
30-49	45.4	45.8	13.6	41.4	19.2	48.0	40.1	13.0	41.8	14.3	19.5	50.0	0.0	3.1	15.9
50+	20.3	0.0	12.7	10.1	51.1	40.0	21.1	13.1	24.4	14.3	30.8	0.0	0.0	1.5	12.3
Missing	0.0	0.0	0.02	0.0	0.0	0.0	0.0	0.05	0.0	0.0	0.0	0.0	0.0	0.0	0.03
Total	100.0	100.0	100.0	100.0	100.0	100.0	100.0	100.0	100.0	100.0	100.0	100.0	100.0	100.0	100.0
